# NADPH Oxidase: A Potential Target for Treatment of Stroke

**DOI:** 10.1155/2016/5026984

**Published:** 2016-01-31

**Authors:** Li Zhang, Jie Wu, Xiaochun Duan, Xiaodi Tian, Haitao Shen, Qing Sun, Gang Chen

**Affiliations:** ^1^Department of Neurosurgery, The First Affiliated Hospital of Soochow University, 188 Shizi Street, Suzhou 215006, China; ^2^Department of Neurosurgery, Zhangjiagang First People's Hospital, No. 68, Jiyang West Road, Zhangjiagang 215699, China; ^3^Department of Neurosurgery, Suzhou Integrated Traditional Chinese and Western Medicine Hospital, 39 Xiashatang Street, Wuzhong 215101, China; ^4^Department of Neurosurgery, Yangzhou No. 1 People's Hospital, No. 45, Taizhou Road, Yangzhou 225001, China

## Abstract

Stroke is the third leading cause of death in industrialized nations. Oxidative stress is involved in the pathogenesis of stroke, and excessive generation of reactive oxygen species (ROS) by mitochondria is thought to be the main cause of oxidative stress. NADPH oxidase (NOX) enzymes have recently been identified and studied as important producers of ROS in brain tissues after stroke. Several reports have shown that knockout or deletion of NOX exerts a neuroprotective effect in three major experimental stroke models. Recent studies also confirmed that NOX inhibitors ameliorate brain injury and improve neurological outcome after stroke. However, the physiological and pathophysiological roles of NOX enzymes in the central nervous system (CNS) are not known well. In this review, we provide a comprehensive summary of our current understanding about expression and physiological function of NOX enzymes in the CNS and its pathophysiological roles in the three major types of stroke: ischemic stroke, intracerebral hemorrhage, and subarachnoid hemorrhage.

## 1. Introduction

Stroke, also known as a cerebrovascular accident, is a group of symptoms in which common clinical features include sudden onset and focal neurological deficits. It is an acute cerebrovascular event closely related to injury of brain tissues because of insufficient regional cerebral perfusion due to a sudden block of a cerebral artery that supplies blood to the brain. The most common causation of stroke is occlusion of a cerebral artery (ischemic stroke accounts for about 87% of all strokes), with only a small part of stroke caused by rupture of the cerebral blood vessels (intracerebral hemorrhage, approximately 10%, and subarachnoid hemorrhage, around 3%, in all cases) [1]. Under heart diseases and cancer, stroke has been third leading cause of death in industrialized nations, and the World Health Organization reports that approximately 15 million people suffer from a stroke every year. Stroke kills up to 5.5 million people annually and cause permanent disability in another 5 million patients [2–4].

In recent years, oxidative stress has attracted considerable attention. It is involved in inflammation, neuronal apoptosis, and necrosis and plays an important role in brain injury after stroke [5–7]. The most important factor for oxidative stress is reactive oxygen species (ROS), which include a variety of small molecule radicals, and the major source of ROS is NADPH oxidase (NOX) [8]. Several NOX subtypes are widely distributed in the cerebral tissues and vasculature. Therefore, the implications of NOX enzymes in cerebrovascular pathology, such as stroke, have received wide attention and have been substantially investigated [3]. One of the important causes of brain damage following stroke is excessive generation of ROS [9]. Moreover, increasing evidences suggest that NOX enzymes play a mechanistic role in the process of brain injury after stroke [10–12].

In this review, we provide a comprehensive description of current knowledge about NOX enzymes in stroke. In the first section, we describe the structure and function of NOX enzymes and the expression of NOX enzymes in the CNS under physiological conditions. At the same time, we summarize the possible mechanisms and roles of NOX enzymes in three stroke pathologies: ischemic stroke, intracerebral hemorrhage, and subarachnoid hemorrhage.

## 2. NOX

### 2.1. What Is NOX?

NOX may refer to either NADPH oxidase [13] or nonphagocytic cell oxidase [14]. The former emphasizes the type of enzyme, and the latter is restricted by cell type. In this review, we discuss only the former.

Nicotinamide adenine dinucleotide phosphate oxidase (NADPH oxidase) was first found in neutrophils and macrophages, so it is also known as phagocyte oxidase (phox). Production of ROS by NADPH oxidase in these two cell types when they undergo an “oxidative burst” during inflammation constitutes the body's defense to pathogens [8]. NOX is localized in the cell membrane, with cytochrome c and flavin adenine dinucleotide (FAD) radicals [14]. The enzyme is composed of six subunits, p22^phox^, p47^phox^, gp91^phox^, p67^phox^, p40^phox^, and the small GTPase Rac. gp91^phox^ and p22^phox^ subunits are located in the plasma membrane and can form active NOX complex when combined with several other cytosolic subunits; gp91^phox^ is the primary functional subunit (catalytic subunit) [15]. In phagocytic cells, NOX is usually in an inactive state. When phagocytic cells are stimulated by extracellular signals, such as hormones, cytokines, bacteria, and other substances, p47^phox^, p67^phox^, p40^phox^, and Rac in the cytosolic subunit can combine with p22^phox^ through its proline-rich tail and form an enzyme complex. This combination changes the conformation of gp91^phox^, induces electronic transmembrane rotation, and activates the enzyme complex which plays a biological role [8, 16].

NOX is classically considered as a key part of electron transport chain in the plasma membrane. It can generate free radical oxidation by reducing one electron in molecular oxygen and produce a series of secondary products (including superoxide, hydroxyl radical, hydrogen peroxide, sodium hypochlorite, ozone, and singlet oxygen) based on the free radical oxidation, with these products referred to as reactive oxygen species (ROS) [8]. ROS play a bactericidal role in phagocytic vesicles and participate in host immunity. This is considered the primary mechanism by which the phagocytic cells kill invading pathogenic microorganisms [16, 17].

In recent years, researchers have found that the following NADPH oxidase subunits, which include all subunits except p40^phox^, are expressed in the nonphagocytic cells: gp91^phox^, p47^phox^, p22^phox^, and p67^phox^ [18]. At present, studies have reported that NADPH oxidase has seven isozymes, nonphagocytic cell oxidases 1/2/3/4/5 (NOX1/2/3/4/5) and dual-function oxidases 1/2 (DUOX1/2), in nonphagocytic cells, which are also part of the NOX (nonphagocytic cell oxidase) family [14]. In nonphagocytic cells, NOX subunits are present in the cytoplasm, and each subunit of the oxidase enzyme is assembled into the functional state for producing ROS. NOX in nonphagocytic cells maintain sustained low levels of activity, even without extracellular stimulation, and constantly produce O_2_^−^. NOX utilize NADH or NADPH as electron donor to produce ROS in nonphagocytic cells [8]. O_2_^−^ produced by NADPH in nonphagocytic cells is generated primarily in the cytoplasm and is involved in the physiological and pathological processes of gene expression, cell proliferation, and apoptosis [18].

ROS are not only by-product of mitochondrial oxidative phosphorylation but also generated by a variety of other sources in cells [13, 14]. The discovery of homologs of the NOX family in the plasma membrane of nonphagocytic cells provides direct evidence for nonphagocytic cellular ROS generation and function and changes our traditional understanding of ROS [19]. It has been widely recognized that ROS from the plasma membrane are produced not only in phagocytic cells but also in many nonphagocytic cells, such as neurons, digestive tract epithelial cells, vascular endothelial cells, mesangial cells, fibroblasts, thyroid cells, and many other cells. ROS participate in host defense and act as messengers in regulation of biological functions in cells [8]. Increasing evidence indicates that ROS act as second messenger and are involved in cell differentiation, regulation of cell proliferation, apoptosis, signal transduction, immune response, and hormone biosynthesis [20–22].

### 2.2. Expression and Function of NOX Enzymes in the CNS

The family of NOX enzymes is widely expressed in all regions of the CNS [23], as shown in both* in vivo* (total brain tissues) and* in vitro* (primary cultured cells) studies. In these studies, researchers detect the expression of all NOX isoforms using a variety of techniques including reverse transcription polymerase chain reaction (RT-PCR), real-time PCR, western blot,* in situ* hybridization, immunohistochemistry, and immunofluorescence staining. Several NOX homologs are coexpressed in the same tissue and cell, although they may have different functions. The results of PCR showed that mRNAs encoding NOX1, NOX2, NOX3, and NOX4 were detected in total brain tissues but not in defined cerebral regions [23]. However, no research provides systematic information on functional protein expression of all NOX isoforms in tissues and cells in the CNS. To our knowledge, there are several studies on NOX2 and NOX4 protein expression in tissues in the CNS [10, 24, 25], and two studies report that NOX1 protein is expressed in primary neurons [26] and astrocytes [27]. At present, there is little data on NOX3 and NOX5 protein expression in the CNS.

In contrast, NOX1, NOX2, NOX4, p22^phox^, p47^phox^, and p67^phox^ mRNAs [28], as well as NOX1, NOX2, NOX4, and p22^phox^ proteins [29, 30], were detected on cerebral vasculature. Interestingly, the expression level of NOX4 in the basilar artery of male rats is higher than that in female rats [29], and gender differences are also observed in expression of other NOX enzymes. It is still not known whether these gender differences in expression of NOX enzymes are functionally important or simply a reflection of gender. Endothelial cells (ECs) show high expression level of NOX1, NOX2, NOX4, p47^phox^, and p67^phox^ in rat cerebral vascular [31]. Endothelial arteries are usually surrounded by pericytes in small cerebral arterioles and capillaries. However, no report has examined NOX enzymes expression in pericytes of cerebral vasculature.

NOX enzymes have been detected in the CNS, but knowledge of their function in normal CNS tissues and cells is limited. This is partly because of the deficiency of powerful tools, and the changes in phenotype are frequently difficult to observe. A study has reported that ROS are implicated in neuronal differentiation during CNS development [32]. NOX enzymes may also play a role in nerve growth factor- (NGF-) induced neuronal differentiation of PC12 cells, and ROS produced by NOX enzymes promotes protein activity and expression that regulate development of neuronal cells [33]. A study has explored the influence of angiotensin II on NOX enzymes in cerebral vasculature [34]. It suggested that NOX2-derived ROS play an essential role in inward Ca^2+^ currents in neurons treated with angiotensin II. Experiments also confirmed that NOX2-derived ROS could affect angiotensin I type receptor (AT1R) signal pathways [35], neuronal activity [36], and CNS regulated cardiovascular functions [37]. As a critical component of the CNS, microglia are resident macrophages that participate in innate immunity in the CNS. Nox family stays in a quiescent status in the absence of stimulation, while be activated in response to several types of stimulations (including damaged or necrotic neuronal cells, pathogens, and abnormal protein aggregation) and releases cytotoxic and inflammatory mediators, for instance, ROS, nitric oxide (NO), and cytokines [38]. The activation of microglia, which is modulated by NOX-derived ROS, is participated in several physiological processes, such as guidance of neuronal cells apoptosis during CNS development [39], inflammatory responses [40], and secretion of neurotransmitters [41]. Thus, NOX enzymes in microglia play important roles in normal physiological functions of the CNS.

## 3. NOX Enzymes in CNS Ischemic and Hemorrhagic Stroke

Several researchers have explored the role of NOX enzymes in excessive production of ROS during progressing of CNS diseases. In this section, after a brief introduction about the respective pathologies, we summarize reports on implications for NOX enzymes from studies of* in vivo* models, as well as possible molecular mechanisms from investigations of* in vitro* systems.

### 3.1. NOX Enzymes in Ischemic Stroke

Ischemic stroke is caused by decreased or blocked blood supply to a certain brain region because of occlusion of a vessel. This occlusion might be thrombotic (caused by blood clots formed* in situ*) or embolic (caused by emboli formed in the heart or another part of the body) [42]. Reducing or interrupting blood flow decreases the supply of oxygen and glucose and prevents the brain from generating ATP, which is required for its enormous energy demands. After ischemic stroke, this energy deficit is most severe in the ischemic core where cell death (including apoptosis and necrosis) occurs rapidly. In addition, a cascade of complex molecular pathways are activated in the neighboring region known as the penumbra [43]. Although the penumbra is functionally impaired, it is potentially salvageable after ischemic stroke [44]. Thus, treatment of ischemic stroke should include repair of the penumbra [45]. Ischemic stroke triggers a series of molecular events beginning with anaerobic glycolysis, lactate acidosis, progressive energy depletion, loss of ability to maintain the membrane potential with depolarization, release of toxic concentration of extracellular glutamate and other excitatory neurotransmitters, activation of gene transcription, inducing protein expression and misfolding aggregation, cellular influx of calcium/sodium and water followed by cell swelling (cytotoxic edema), mitochondrial failure, ROS production, and inflammatory responses, finally leading to brain tissue injury [46–50].

Permanent middle cerebral artery occlusion (pMCAO) and transient middle cerebral artery occlusion (tMCAO) followed by reperfusion are major experimental animal models used to investigate ischemic stroke [51]. It has been reported that NOX2 protein increases from 24 h to 72 h after reperfusion in endothelial cells [52] and microglia [53] of the penumbra in mice pMCAO and tMCAO models. NOX4 has also been confirmed to increase in the brain after ischemic stroke. In a model of MCAO, NOX4 mRNA levels in neurons increase within day 1, peak between days 7 and 15, and slowly decline until day 30. The NOX4 mRNA level in newly formed capillaries increases in parallel with the peak, suggesting that NOX4 plays a role in repair of brain damage [10]. We also found that the mRNA and protein levels of NOX2, NOX4, and DUOX1 increase in neurons, astrocytes, and endothelial cells in a rat MCAO model, but there is no significant change in NOX1, NOX3, and DUOX2 [54]. In analysis of whole brain tissues, the mRNA and protein levels of NOX2 and p22^phox^ increase in the ischemic hemisphere in a rat model of MCAO [55], and NOX4 protein increases in the ischemic cortex and basal ganglia after ischemic stroke in mice [56]. In general, these data indicate that NOX2, NOX4, and DUOX1 expression increase after ischemic stroke. In addition, a study using a model of endothelin-1-induced stroke has reported that NOX activity increases in arteries of the penumbra [57]. To our knowledge, no data are available on NOX5 expression after ischemic stroke, and possible changes in expression of this homolog remain to be examined.

Knockout (KO) animals and specific inhibitors are powerful tools for exploring the roles of different NOX isoforms (Table 1). After ischemia/reperfusion in mice, genetic deletion of NOX2 significantly reduced disruption of blood-brain barrier and infarct size [58]. However, NOX2 is not obviously involved in the pathogenesis of brain damage in newborn pups after hypoxia/ischemia [59, 60]. It has been demonstrated that NOX2 is the major source of ROS after ischemic stroke, not only in circulating leukocytes that have infiltrated the CNS with reperfusion but also in CNS cells [61]. NOX4 has also been shown to have a protective effect on ischemic stroke; NOX4-deficient mice had reduced infarct size and improved neurological outcome after ischemic stroke [56]. Two investigations using NOX1-knockout mice did not show neuroprotection in respective MCAO model [56, 62]. However, in a rat tMCAO model, absence of NOX1 significantly reduced lesion size, improved neurological outcome, preserved blood-brain barrier integrity, and reduced cerebral edema [63]. To our knowledge, no knockout animal models of other NOX subtypes have been used to research experimental stroke.

Several pharmacological studies have confirmed that NOX enzymes contribute to the progression of brain injury after ischemic stroke. In an* in vivo* model of endothelin-1-induced tMCAO, treatment with the NOX inhibitor diphenyleneiodonium (DPI) decreases superoxide production in arteries of the penumbra area and of the contralateral part to the infarct [57]. Another report showed that administration of DPI combined with dimethylsulfoxide (DMSO) reduced infarct size and blood-brain barrier disruption [64]. In addition, treatment with the NOX inhibitor apocynin before ischemia also exerted a neuroprotective effect, resulting in reduced infarct volume and improved neurological outcome [60, 65]. It was also reported that apocynin inhibits the activity of matrix metalloproteinase-9 (MMP-9), improving the blood-brain barrier disruption and ameliorating neurological outcome [66]. Honokiol, another inhibitor of NOX, has also been shown to have a protective effect on cerebral ischemia-reperfusion injury by inhibiting ROS generation and reducing lesion size [67]. Other NOX inhibitors have not been studied in stroke models, and the safety and specificity of these inhibitors remain challenging for their clinical application.

To investigate the mechanisms of cellular death after ischemic stroke, primary neurons treated with oxygen/glucose deprivation and reoxygenation were used as an* in vitro* model of ischemic stroke. Results suggest that NOX2 contributes to generation and accumulation of ROS in neurons and neuronal death in the reoxygenation phase. These effects were blocked by NADPH oxidase inhibitors and were absent in neurons from gp91^phox^ knockout mice [68]. Meanwhile, genetic absence of p47^phox^, which is essential for NOX2 activation, also exhibits neuroprotective effects (postischemic superoxide production and cell death were prevented) in cultured neurons [69].

### 3.2. NOX Enzymes in Hemorrhagic Stroke

Hemorrhagic strokes are usually divided into intracerebral hemorrhage (ICH) or subarachnoid hemorrhage (SAH). In general, hemorrhagic stroke causes a higher mortality and morbidity than ischemic stroke [70]. ICH is often caused by hypertension, bleeding disorders, or amyloid angiopathy, while SAH may be caused by trauma or rupture of an aneurysm [71, 72]. The causes of brain injury after ICH are complex. Initial damage is due to the mechanical force induced by formation of the hematoma. Hematoma expansion, edema, and inflammation cause subsequent damage in brain tissues [73]. Excitatory amino acids toxicity is a major factor in secondary brain injury after ICH and glutamate is the primary excitatory neurotransmitter [74]. Meanwhile, the major and frequent complications of aneurysmal SAH are early brain injury [75] and cerebral vasospasm [76].

A previous study has shown that, in a mouse model of ICH, mRNA levels of gp91^phox^ subunit were significantly increased, and immunohistochemistry showed that gp91^phox^ subunit expression levels were also significantly upregulated. In a collagenase-induced ICH model, gp91^phox^ knockout mice displayed significant decreases in the volume of hematoma, brain edema, neurological deficit, and mortality compared with wild-type ICH mice. These data indicate that NOX2 plays an important role in brain damage due to ICH [77]. The activity of NOX enzymes increased significantly in hypertensive mice with spontaneous ICH [78]. A study also reported that activity of NADPH-d increased significantly in the rat striatum after ICH [79]. However, another study found that treatment with apocynin did not ameliorate the outcome of rats after ICH. The hemorrhage volume, brain edema, and neurologic score were not impaired in rats treated with different doses of apocynin (3–30 mg/kg) [80]. This discrepancy may be due to the dose of apocynin. Several investigations have reported that low doses of apocynin show benefit effects, but high doses (more than 3.75 mg/kg) increase intracerebral hemorrhage and mortality of experimental animals [60, 81].

Several studies have demonstrated the roles of NOX enzymes in SAH models. With increased expression levels of NOX2, oxidative stress [82], cerebral vasospasms [30], and neurological deficits [83] obviously increased after SAH. However, when SAH rats were treated with the inhibitors of NOX2, DPI [82], or apocynin [83], these neuronal damage types were improved significantly. It has been reported that reduced neuronal damage observed after treatment of SAH with hyperbaric oxygen may involve downregulation of NOX2 expression [84]. Further research, using a rat model of SAH, confirmed that mRNA levels of gp91^phox^ subunit and NOX activity were significantly increased and that hyperbaric oxygen exerts neuroprotection by inhibiting these changes [85]. However, hyperbaric oxygen is not a specific inhibitor for NOX2 and no NOX2-knockout animals were used in these studies. Thus, these results should be considered preliminary. To our surprise, there is no reduction in mortality rate, brain-water content, and intensity of oxidative stress in NOX2-deficient mice after SAH [86]. To the best of our knowledge, no other NOX isoforms deficient animals were used in models of SAH.

## 4. Conclusions

**Figure 1:**
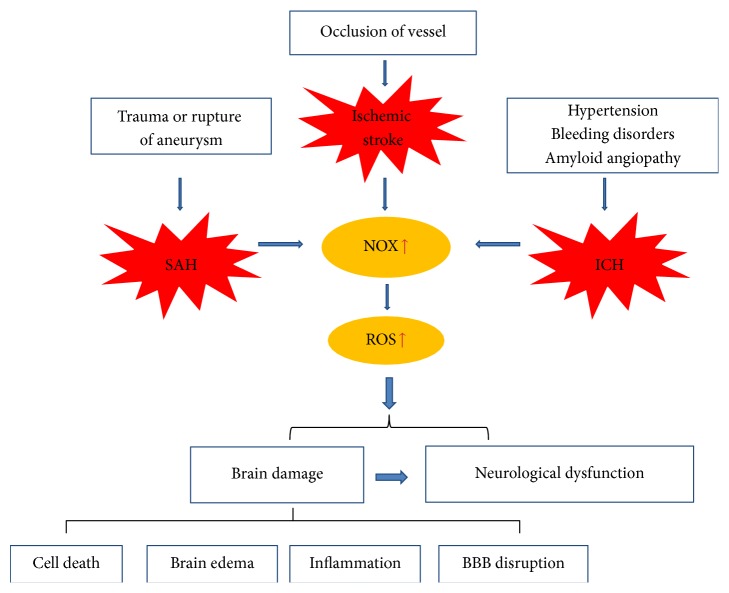
The role of NADPH oxidase enzymes in brain damage and neurological dysfunction after stroke. ICH: intracerebral hemorrhage; SAH: subarachnoid hemorrhage; ROS: reactive oxygen species; BBB disruption: blood-brain barrier disruption.

Oxidative stress contributes to brain damage after stroke, and NADPH oxidase enzymes are a major source of ROS in this context (Figure 1). NOX enzymes are broadly distributed in the CNS, including neurons, astrocytes, microglia, and the cerebral vasculature. The expression of several NOX subtypes significantly increases in brain tissue and cerebral vasculature after stroke. Genetic absence or pharmacological inhibition of functional NADPH oxidases, especially NOX2 and NOX4, reduces brain tissue damage and improves neurological outcome following experimental stroke. Thus, inhibition of NADPH oxidase might be an effective strategy for stroke therapy. The use of knockout animal models and the development of inhibitors that target specific NOX homologs will further increase our understanding of the roles that NOX enzymes play in stroke. Moreover, a clear understanding of the regulation of ROS producing systems, their distribution in the cytoplasm and organelles, and mechanisms of activation of NOX enzymes in brain cells and cerebral vasculature under pathophysiological conditions will contribute to better stroke therapy.

## Figures and Tables

**Table 1:** Published studies on genetic absence or pharmacological inhibition of NOX in stroke.

NOX isoform	Stroke model	Genetic absence or pharmacological inhibitor	Parameters analyzed	Conclusion	Reference
NOX1	tMCAO and pMCAO	NOX1−/−	Lesion size, neurological outcome, BBB integrity, cerebral edema	NOX1 KO has a protective effect	[63]

NOX1	tMCAO	NOX1−/−	Infarct volume, cerebral edema, neurological outcome	NO neuroprotection	[62]

NOX1	tMCAO and pMCAO	NOX1−/−	Infarct volume, neurological outcome, BBB integrity, cerebral edema, ROS, RNS, apoptosis	NO neuroprotection	[56]

NOX2	tMCAO	NOX2−/−	Infarct volume, ROS	NOX2 KO has a protective effect	[61]

NOX2	tMCAO	NOX2−/−	Infarct volume, BBB integrity	NOX2 KO has a protective effect	[58]

NOX2	tMCAO	NOX2−/−	Mortality, infarct volume, neurological outcome, ROS	NOX2 KO has a protective effect	[60]

NOX2	tMCAO and pMCAO	NOX2−/−	Infarct volume, neurological outcome, BBB integrity, cerebral edema, ROS, RNS, apoptosis	NO neuroprotection	[56]

NOX2	ICH	NOX2−/−	Mortality, hematoma volume, neurological deficit, brain edema	NOX2 KO has a protective effect	[77]

NOX2	SAH	NOX2−/−	Mortality, brain edema, oxidative stress	NO neuroprotection	[86]

NOX4	tMCAO and pMCAO	NOX4−/−	Infarct volume, neurological outcome, BBB integrity, cerebral edema, ROS, RNS, apoptosis	NOX4 KO has a protective effect	[56]
	tMCAO	DPI	Superoxide production level	DPI has a protective effect	[57]
	tMCAO	DPI and DMSO	Infarct volume, neurological outcome, BBB integrity, MMP-2/ MMP-9 activity	DPI and DMSO exert neuroprotection	[64]
	tMCAO	Apocynin	Infarct volume, neurological outcome	Apocynin has a protective effect	[65]
	tMCAO	Apocynin	Neurological outcome, BBB integrity, MMP-9 activity	Apocynin has a protective effect	[66]
	tMCAO	Honokiol	Lesion size, ROS, neutrophil activation/infiltration, calcium influx	Honokiol has a protective effect	[67]
	ICH	Apocynin	Hemorrhage volume, brain edema, neurological outcome	NO neuroprotection	[80]
	SAH	DPI	ROS, autoregulatory vasodilation	DPI has a protective effect	[82]
	SAH	Apocynin	cerebral vasospasm, superoxide level, neurological deficit	Apocynin has a protective effect	[83]

tMCAO: transient middle cerebral artery occlusion; pMCAO: permanent middle cerebral artery occlusion; BBB integrity: blood-brain barrier integrity; KO: knockout; ROS: reactive oxygen species; RNS: reactive nitrogen species; ICH: intracerebral hemorrhage; SAH: subarachnoid hemorrhage; DPI: diphenyleneiodonium; DMSO: dimethylsulfoxide; MMP-2/MMP-9: matrix metalloproteinase-2/matrix metalloproteinase-9.
